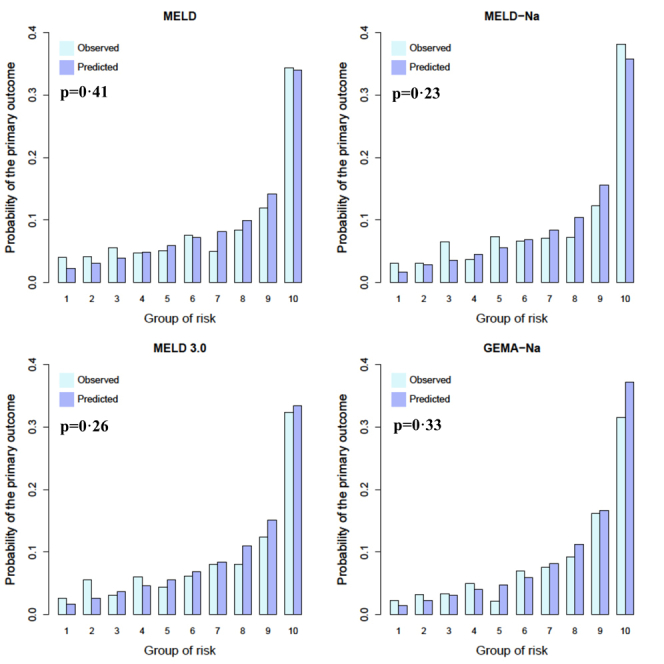# GEMA-Na and MELD 3.0 severity scores to address sex disparities for accessing liver transplantation: a nationwide retrospective cohort study

**DOI:** 10.1016/j.eclinm.2024.102737

**Published:** 2024-07-18

**Authors:** Manuel Luis Rodríguez-Perálvarez, Gloria de la Rosa, Antonio Manuel Gómez-Orellana, María Victoria Aguilera, Teresa Pascual Vicente, Sheila Pereira, María Luisa Ortiz, Giulia Pagano, Francisco Suarez, Rocío González Grande, Alba Cachero, Santiago Tomé, Mónica Barreales, Rosa Martín Mateos, Sonia Pascual, Mario Romero, Itxarone Bilbao, Carmen Alonso Martín, Elena Otón, Luisa González Diéguez, María Dolores Espinosa, Ana Arias Milla, Gerardo Blanco Fernández, Sara Lorente, Antonio Cuadrado Lavín, Amaya Redín García, Clara Sánchez Cano, Carmen Cepeda-Franco, José Antonio Pons, Jordi Colmenero, David Guijo-Rubio, Alejandra Otero, Alberto Amador Navarrete, Sarai Romero Moreno, María Rodríguez Soler, César Hervás Martínez, Mikel Gastaca

**Affiliations:** a Department of Hepatology and Liver Transplantation, Hospital Universitario Reina Sofía, IMIBIC, Avda. Menéndez Pidal s/n, 14014, Córdoba, Spain; b Centro de Investigación Biomédica en Red de Enfermedades Hepáticas y Digestivas (CIBERehd), Monforte de Lemos 3-5, 28029, Madrid, Spain; c Organización Nacional de Trasplantes (ONT), Sinesio Delgado, 8, Fuencarral-El Pardo, 28029, Madrid, Spain; d Department of Computer Science and Numerical Analysis, Universidad de Córdoba, Escuela Politécnica Superior de Córdoba, IMIBIC, Campus Universitario de Rabanales, Albert Einstein Building, Ctra. N-IV, Km. 396, 14071, Córdoba, Spain; e Department of Hepatology and Liver Transplantation, Hospital La Fe e Instituto de Investigación sanitaria La Fe, Avenida de Fernando Abril Martorell, 106, 46026, Valencia, Spain; f Department of HPB surgery and Liver Transplantation, Hospital Universitario de Cruces, Plaza de Cruces, S/N, 48903, Barakaldo, Bilbao, Spain; g Department of HPB surgery and Liver Transplantation, Hospital Virgen del Rocío, Av. Manuel Siurot, s/n, 41013, Sevilla, Spain; h Department of Hepatology and Liver Transplantation, Hospital Universitario Virgen Arrixaca, Ctra. Madrid-Cartagena, s/n, 30120, Murcia, Spain; i Department of Hepatology and Liver Transplantation, Hospital Clinic, IDIBAPS, C/ Villarroel, 170, 08036, Barcelona, Spain; j Department of Hepatology and Liver Transplantation, Centro Hospitalario Universitario de A Coruña, Jubias De Arriba 82, 15006, A Coruña, Spain; k Department of Hepatology and Liver Transplantation, Hospital Regional Universitario de Málaga, Avenida Carlos de Haya, s/n, 29001, Málaga, Spain; l Department of Liver Transplantation, Hospital Universitario de Bellvitge, Carrer De La Feixa Llarga, S/n, 08907, Hospitalet De Llobregat, Spain; m Department of Liver Transplantation, Centro Hospitalario Universitario de Santiago, Calle da choupana, 15706, Santiago de Compostela, Spain; n Department of Hepatology and Liver Transplantation, Hospital Universitario 12 de Octubre, Av de Córdoba, s/n, 28041, Madrid, Spain; o Department of Liver Transplantation, Hospital Universitario Ramón y Cajal, IRYCIS, Universidad de Alcalá, Calle de Antoniorrobles, 1, 28034, Madrid, Spain; p Department of Hepatology and Liver Transplantation, Hospital General Universitario Dr. Balmis de Alicante, ISABIAL, Av. Pintor Baeza, 12, 03010, Alicante, Spain; q Department of Hepatology and Liver Transplantation, Hospital General Universitario e Instituto de Investigación Biomédica Gregorio Marañón, Calle Doctor Esquerdo, 46, 28007, Madrid, Spain; r Department of Liver Transplantation, Hospital Universitario Vall d’Hebron, VHIR, Pg.de la Vall d'Hebron 119, Barcelona, Spain; s Department of Hepatology and Liver Transplantation, Hospital Rio Hortega, Calle La Dulzaina, 2, 47012, Valladolid, Spain; t Department of Hepatology and Liver Transplantation, Hospital Virgen de la Candelaria, Carretera Del Rosario, 145, 38010, Santa Cruz de Tenerife, Spain; u Department of Hepatology and Liver Transplantation, Hospital Universitario Central de Asturias, Avenida de Roma, s/n, 33011, Oviedo, Spain; v Department of Hepatology and Liver Transplantation, Hospital Virgen de las Nieves, Avenida de las Fuerzas Armadas, 2, 18014, Granada, Spain; w Department of Hepatology and Liver Transplantation, Hospital Universitario Puerta de Hierro, Calle Manuel de Falla, 1, 28222, Madrid, Spain; x Department of Liver Transplantation, Hospital Universitario de Badajoz, Avenida de Elvas s/n, 06071, Badajoz, Spain; y Department of Hepatology and Liver Transplantation, Hospital Universitario Lozano Blesa, Instituto de Investigaciones Sanitarias de Aragón (IIS Aragón), Avenida San Juan Bosco, 15, 50009, Zaragoza, Spain; z Department of Hepatology and Liver Transplantation, Hospital Universitario Marqués de Valdecilla, IDIVAL, Avenida Valdecilla, 25, 39008, Santander, Spain; aa Department of Hepatology, HPB surgery and Liver Transplantation, Clínica Universidad de Navarra, IdiSNA, Avda. Pío XII, 36, 31008, Pamplona, Spain; ab Department of Signal Processing and Communications, Universidad de Alcalá, Plaza De San Diego, S/n, 28801, Alcalá De Henares, Madrid, Spain

**Keywords:** Sex, Equity, Liver transplantation, Urgency, Allocation

## Abstract

**Background:**

The Gender-Equity Model for liver Allocation corrected by serum sodium (GEMA-Na) and the Model for End-stage Liver Disease 3.0 (MELD 3.0) could amend sex disparities for accessing liver transplantation (LT). We aimed to assess these inequities in Spain and to compare the performance of GEMA-Na and MELD 3.0.

**Methods:**

Nationwide cohort study including adult patients listed for a first elective LT (January 2016–December 2021). The primary outcome was mortality or delisting for sickness within the first 90 days. Independent predictors of the primary outcome were evaluated using multivariate Cox's regression with adjusted relative risks (RR) and 95% confidence intervals (95% CI). The discrimination of GEMA-Na and MELD 3.0was assessed using Harrell c-statistics (Hc).

**Findings:**

The study included 6071 patients (4697 men and 1374 women). Mortality or delisting for clinical deterioration occurred in 286 patients at 90 days (4.7%). Women had reduced access to LT (83.7% vs. 85.9%; p = 0.037) and increased risk of mortality or delisting for sickness at 90 days (adjusted RR = 1.57 [95% CI 1.09–2.28]; p = 0.017). Female sex remained as an independent risk factor when using MELD or MELD-Na but lost its significance in the presence of GEMA-Na or MELD 3.0. Among patients included for reasons other than tumours (n = 3606; 59.4%), GEMA-Na had Hc = 0.753 (95% CI 0.715–0.792), which was higher than MELD 3.0 (Hc = 0.726 [95% CI 0.686–0.767; p = 0.001), showing both models adequate calibration.

**Interpretation:**

GEMA-Na and MELD 3.0 might correct sex disparities for accessing LT, but GEMA-Na provides more accurate predictions of waiting list outcomes and could be considered the standard of care for waiting list prioritization.

**Funding:**

10.13039/501100004587Instituto de Salud Carlos III, 10.13039/501100011033Agencia Estatal de Investigación (Spain), and 10.13039/501100000780European Union.

**Research in context**

**Evidence before this study**

Women wait longer to receive a liver graft and they are more likely to die on the waiting list or to be excluded due to clinical deterioration but two novel scores, GEMA-Na and MELD 3.0, have been developed to address sex inequities. We searched MEDLINE, EMBASE and Science Citation Index databases from inception to December 2023, for studies evaluating the performance of MELD 3.0, GEMA-Na, or both, to predict waiting list outcomes among liver transplant candidates. We used different combinations of the following keywords or equivalent free-text terms, without language restrictions (“gender” OR “sex” OR “women” OR “disparities”) AND (“waiting list”) AND (“liver transplantation”) AND (“MELD 3.0” OR “GEMA-Na”). In their respective original studies, both MELD 3.0 and GEMA-Na showed improved discrimination to predict 90-days mortality or delisting for sickness than MELD-Na but a direct comparison between the two scores was evaluated only in two relatively small cohorts with a wide recruitment period.

**Added value of this study**

This is the first nationwide study evaluating sex disparities for accessing liver transplantation in Europe, and particularly in a setting of high deceased donation rates and short waiting time for liver transplantation. Women were less likely to receive a liver graft than men and they had 57% excess risk of mortality or delisting for sickness at 90 days after adjusting for potential confounders. Female sex remained as an independent risk factor when using MELD or MELD-Na but lost its significance when employing GEMA-Na or MELD 3.0. GEMA-Na made more accurate predictions of mortality or delisting for sickness within the first 90 days than MELD 3.0.

**Implications of all the available evidence**

Both GEMA-Na or MELD 3.0 may be equally effective to correct sex disparities for accessing liver transplantation, but GEMA-Na produces more accurate predictions of mortality or delisting for sickness. Unless future evidence proves otherwise, GEMA-Na could be considered the standard of care for liver transplant waiting list prioritization.

## Introduction

The sickest-first policy for liver allocation has prevailed over decades since the creation of the Model for End Stage Liver Disease (MELD) and its sodium-corrected variant (MELD-Na).^1^ Patients with increased likelihood of short-term mortality or clinical deterioration beyond transplant suitability are granted the first positions on the waiting list for an earlier access to liver transplantation (LT).^2^ The implementation of MELD created sex-based disparities for accessing LT which were not previously present.^3^ Compared to their male counterparts, women wait longer to receive a liver graft and they are more likely to die on the waiting list or to be excluded for sickness.^4^^,^^5^ Serum creatinine is considered the main factor underlying sex disparities. Indeed, women show reduced muscle mass in average than men and receive less creatinine-derived MELD points.^6^ In addition, the severity of patients with acute alcohol-associated hepatitis or acute-on-chronic liver failure may be underestimated by MELD and MELD-Na,^7^ offering opportunities for novel scores to make more accurate and equitable predictions.

MELD 3.0 is a relevant update of MELD-Na which has incorporated sex and serum albumin as new covariates, controlled relevant interactions, and capped serum creatinine at 3.0 mg/dL. MELD 3.0 showed improved discrimination than MELD-Na, and assigned 1.3 extra points to women. These features supported its recent adoption in the United States.^8^ The Gender-Equity Model for Liver Allocation corrected by serum sodium (GEMA-Na)^9^ replaced serum creatinine with the Royal Free glomerular filtration rate (RFH-GFR),^10^ with reweighting and refitting of the remaining components of MELD-Na. GEMA-Na was trained and internally validated in the United Kingdom, and externally validated in Australia, where it consistently showed improved discrimination over MELD-Na and MELD 3.0.^9^ The clinical benefit was more pronounced in women and these results were confirmed in a cohort of the Lazio region in Italy.^11^ However, the widespread implementation of novel prioritization models requires further validation.

Spain holds the highest deceased donation rates worldwide with 48.9 donors per million habitants.^12^ The median length on the waiting list for elective LT was only 55 days in 2022 and mortality rates on the waiting list are lower than in other European countries, United States or Canada. The utility of urgency-based prioritization scores in this context has not been demonstrated and it is unclear whether the implementation of novel scores such as GEMA-Na or MELD 3.0 would make a meaningful impact on waiting list outcomes or in addressing sex disparities.

The aims of the present study were to evaluate sex-based disparities for accessing LT in Spain and to compare the performance of the newly created scores, MELD 3.0 and GEMA-Na, to amend such inequities.

## Methods

### Study population, data source and ethical considerations

This is a nationwide cohort study including all adult patients who entered the waiting list for elective LT in Spain from 1st January 2016 to 31st December 2021. Exclusion criteria were as follows: acute liver failure listed for urgent LT, living donor, combined organ transplantation, re-transplantation, or impossibility to calculate predicting scores due to missing analytical values. Patients were followed until transplantation, death, or removal from the waiting list, whichever occurred first. Database closure was on 30th June 2023 to ensure a complete registration of waiting list events. The data was obtained from the *Organización Nacional de Trasplantes* (ONT), which is the official organ attached to the Spanish Ministry of Health responsible for the obtention and clinical use of organs, tissues, and cells with transplant purposes. The ONT database contains precise information of all LT institutions in Spain which contribute with prospectively recorded information regarding patient demographics, aetiology of liver disease, reason for inclusion on the waiting list, date of inclusion, and outcomes on the waiting list. Analytical values required for the calculation of MELD, MELD-Na, MELD 3.0, and GEMA-Na were obtained from each patient's electronical medical record.

### Ethics

The study complies with the Declaration of Helsinki and was approved by the Andalusian ethics committee (reference 5408, 2022). The need of informed consent was waived by the ethics committee.

### Definitions, variables, and predicting scores

Age, sex assigned at birth, height, weight, aetiology of liver disease, reason for being included on the waiting list, date of inclusion, events on the waiting list, and date of such events were retrieved from the ONT database. The indication for waiting list inclusion was categorized as follows: a) hepatic insufficiency, meaning a severe impairment of synthetic liver function with or without clinical decompensations; b) refractory ascites, including both, diuretic-resistant or diuretic-intractable ascites requiring large volume paracentesis (≥5 L per session) at least every 4 weeks^13^; c) recurrent or persistent hepatic encephalopathy^14^; d) hepatocellular carcinoma (HCC); e) other tumours apart from HCC; and f) non-tumoral special indications not falling into previous categories. Moderate-severe ascites was considered if the patient required a large volume paracentesis within the 4 weeks prior to waiting list inclusion or if it was clinically evident in the physical examination and was confirmed in imaging techniques.^13^ Since an abdominal doppler ultrasound and/or angio-computed tomography may be routinely performed as part of the pre-LT workup, these criteria ensured an objective evaluation of ascites and avoided biased assessments. The following generalized addictive Cox's regression models were calculated: MELD, MELD-Na, MELD 3.0, and GEMA-Na. A calculator for MELD, MELD-Na, and GEMA-Na is available at: https://en.gemascore.com/. A calculator for MELD 3.0 is available at: https://medcalculators.stanford.edu/meld Detailed information regarding the calculation of predicting scores is described in the appendix.

### Outcomes and sensitivity analyses

The primary outcome of the study was mortality or exclusion from the waiting list due to clinical deterioration beyond transplant suitability within the first 90 days as a time-dependent event, aligning with previous studies.^1^^,^^8^^,^^9^ Outcome data was right censored at 90 days post-inclusion, or earlier than that if the patient underwent LT or was excluded for reasons other than clinical worsening.

The analysis was performed in the overall cohort and sensitivity analyses were performed in pre-defined subgroups of interest, namely women, height <160 cm, presence of ascites, and LT indication: a) non-tumoral indications; b) decompensated cirrhosis, which comprised hepatic insufficiency, refractory ascites, and recurrent/persistent hepatic encephalopathy; and c) hepatic insufficiency alone.

### Performance of the models

The sample size requirement was aimed to detect clinically meaningful differences between the discrimination capacity of GEMA-Na and MELD 3.0 to predict the primary outcome. We used a method specifically designed to compare predicting scores with right-censored outcomes.^15^ Under a statistical power of 90% and alpha error of 0.05, the minimum sample size required was 4932 patients. More details about sample size calculation are provided as supplementary material. MELD, MELD-Na, MELD 3.0, and GEMA-Na were assessed in terms of discrimination, calibration, reclassification, and differential prioritization. Discrimination refers to the ability of the model to differentiate between patients experiencing or not the primary outcome. Discrimination was assessed using the Hc statistic, which is specific for time dependent outcomes with right-censoring. The statistical comparison of discrimination among different models was performed using a one-shot nonparametric approach which does not require resampling as described by Kang et al.^16^ The Brier score was used to measure the overall accuracy of the scores. Calibration informs about the homogeneity of the predictions across the disease severity spectrum. The Greenwood-Nam-D’Agostino test, which assess the goodness-of-fit after stratifying the population in deciles of risk, was used for calibration analyses.^17^ Reclassification visualizes the impact of transitioning from one model to another on the waiting list composition through the proportion of patients with a clinically meaningful change of ≥2 score points. Finally, differential prioritization compared outcomes between the subgroup of patients who would receive a LT only with one model or another. Differential prioritization was assessed in patients with indications other than tumours to better capture the true impact in real clinical practice. For the simulation analysis, patients were ranked according to each score and the available organs within the first 90 days, which equals the number of LT performed within the same period, were allocated commencing from the patient with the highest score downwards. Comparing outcomes of patients differentially prioritized according to each model allowed to estimate the number of potential deaths avoided.^9^

### Statistics

Categorical variables were displayed as absolute number and percentages. Continuous variables were expressed as mean and standard deviations, excepting for those with a skewed distribution, in which median and interquartile range (IQR) were used. The appropriate contrast tests were used according to the type of variables involved in the analysis: chi-square for frequencies, student T test for continuous variables following normal distribution, and Mann–Whitney U test for continuous variables following abnormal distribution. Kaplan–Meier curves (log-rank test) and multivariate Cox's regression were used to analyse predictors of mortality or exclusion from the waiting list due to clinical deterioration. All variables with a p < 0.20 in the univariate analysis entered the initial model. Not significant covariates were removed in a backward stepwise process. Potential confounding factors were identified if their removal from the model motivated a significant change greater than 15% in any of the beta coefficients of the remaining covariates. The final model was composed by significant covariates, confounding factors, and clinically relevant covariates as per authors' judgement. A bilateral p < 0.05 was considered statistically significant. Analyses were performed by using R v4.1.2 (RStudio Inc., Boston, USA) and SPSS 27.0 (IBM, Chicago, USA).

### Role of the funding source

The funding source had no role in the study design, data collection and analysis, manuscript preparation or in the decision to publish the study.

## Results

### Study population and outcomes on the waiting list

**Fig. 1:**
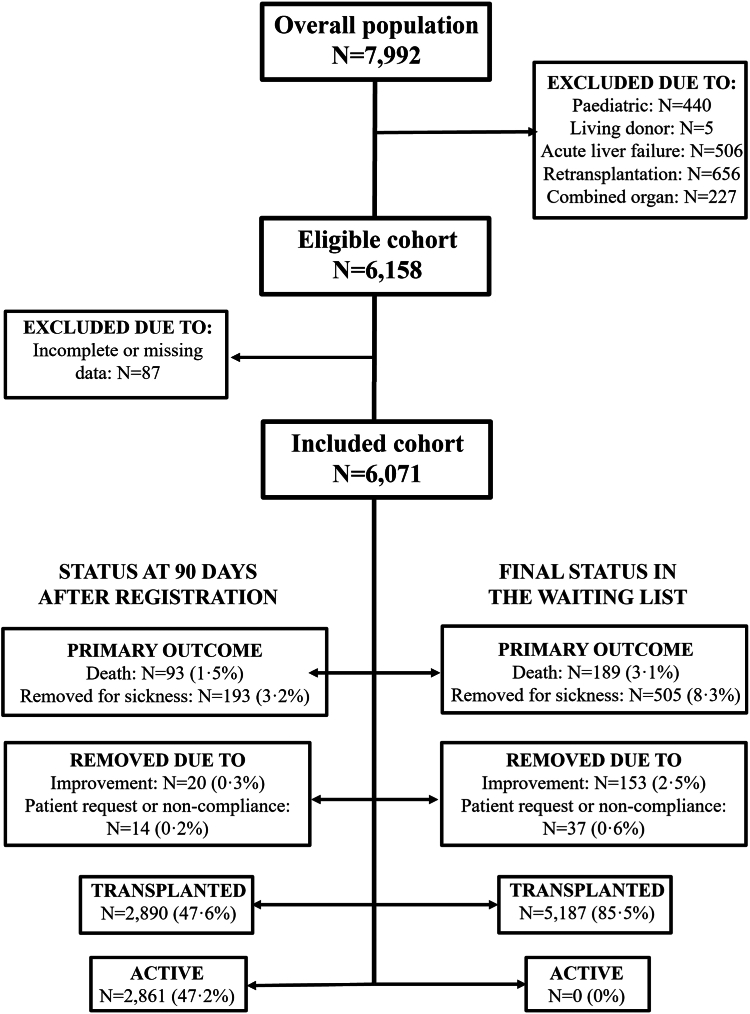
Study flowchart showing outcomes on the waiting list, both at 90 days after registration (left side) and at maximal follow-up (right side).

Fig. 1 shows the study flowchart. The study population comprised 6071 patients (4697 men and 1374 women) who were included on the waiting list for LT in any of the institutions which compose the Spanish LT network (Supplementary Table S1). Table 1 shows the main clinical characteristics of the study population and differences between men and women. The leading indication for LT was HCC in men (43.8%) and hepatic insufficiency in women (34.1%). Women were significantly shorter than men (159.35 ± 7.08 cm vs. 171.07 ± 7.02 cm; p < 0.001) and had reduced body mass index (26.39 ± 5.53 kg/m2 vs. 27.86 ± 4.72 kg/m2; p < 0.001). Women had higher bilirubin (2.05 mg/dL [IQR 1–4.22] vs. 1.80 [IQR 1–3.40]; p < 0.001) and lower albumin (3.40 ± 0.68 g/dL vs. 3.51 ± 0.69; p < 0.001) than men. Noteworthy, despite having lower creatinine (0.84 ± 0.54 mg/dL vs. 0.96 ± 0.47 mg/dL; p < 0.001), women showed worse renal function according to the RFH-GFR compared to men (65.70 ± 25.38 ml/min vs. 69.56 ± 23.54 ml/min). Women and men had comparable MELD and MELD-Na scores, but women obtained higher MELD 3.0 score (16.29 ± 6.44 vs. 14.61 ± 6.65; p < 0.001) and GEMA-Na score (17.02 ± 6.08 vs. 16.09 ± 6.07; p < 0.001). Supplementary Table S2 shows clinical characteristics of patients with indications for LT other than tumours stratified by sex assigned at birth.

**Table 1:** Clinical features of 6071 patients enlisted for a first deceased donor liver transplantation in Spain from 2016 to 2021.

Variable	Overall (N = 6071)	Men (N = 4697)	Women (N = 1374)	p
Age	57.81 ± 8.60	58.26 ± 7.74	56.28 ± 10.88	<0.001
Height (cm)	168.42 ± 8.58	171.07 ± 7.02	159.35 ± 7.08	<0.001
Weight (kg)	78.19 ± 15.85	81.50 ± 14.72	66.85 ± 14.22	<0.001
Body mass index (kg/m2)	27.53 ± 4.95	27.86 ± 4.72	26.39 ± 5.53	<0.001
Aetiology (alcohol)	3026 (49.8%)	2668 (56.8%)	358 (22.6%)	<0.001
Aetiology (hepatitis C)	1325 (21.8%)	1084 (23.1%)	241 (17.5%)	<0.001
Aetiology (hepatitis B)	325 (5.4%)	287 (6.1%)	38 (2.8%)	<0.001
Aetiology (autoimmune)	485 (8%)	166 (3.5%)	319 (23.2%)	<0.001
Aetiology (MASH)	97 (1.6%)	66 (1.4%)	31 (2.3%)	0.027
Aetiology (cryptogenic)	253 (4.2%)	157 (3.3%)	96 (7%)	<0.001
Aetiology (others)	971 (16%)	633 (13.5%)	338 (24.6%)	<0.001
Indication for LT				<0.001
Hepatic insufficiency	1832 (30.2%)	1364 (29%)	468 (34.1%)	
HCC	2371 (39.1%)	2053 (43.8%)	318 (23.2%)	
Refractory ascites	783 (12.9%)	608 (12.9%)	175 (12.7%)	
Chronic encephalopathy	272 (4.5%)	207 (4.4%)	65 (4.7%)	
Other indications (tumoral)	94 (1.5%)	46 (1%)	300 (21.8%)	
Other indications (non-tumoral)	719 (11.8%)	419 (8.9%)	48 (3.5%)	
Use of diuretics at inclusion	3103 (51.1%)	2348 (50%)	755 (54.9%)	0.001
Ascites				0.027
No	2972 (49%)	2331 (49.6%)	641 (46.7%)	
Mild	970 (16%)	725 (15.4%)	245 (17.8%)	
Moderate-severe	2129 (35%)	1641 (35%)	488 (35.5%)	
Urea (mg/dL)	40.73 ± 25.28	41.00 ± 25.56	39.80 ± 24.27	0.120
Creatinine (mg/dL)	0.93 ± 0.49	0.96 ± 0.47	0.84 ± 0.54	<0.001
RFH-GFR (ml/min)	68.69 ± 24.02	69.56 ± 23.54	65.70 ± 25.38	<0.001
International Normalized Ratio	1.43 ± 0.46	1.43 ± 0.46	1.42 ± 0.45	0.372
Bilirubin (mg/dL)	1.80 (IQR 1–3.60)	1.80 (IQR 1–3.40)	2.05 (IQR 1–4.22)	<0.001
Sodium (mmol/L)	137.59 ± 4.77	137.60 ± 4.78	137.54 ± 4.75	0.644
Albumin (g/dL)	3.48 ± 0.69	3.51 ± 0.69	3.40 ± 0.68	<0.001
MELD	13.76 ± 5.76	13.72 ± 5.79	13.88 ± 5.65	0.368
MELD-Na	15.61 ± 6.52	15.56 ± 6.57	15.77 ± 6.36	0.290
MELD 3.0	14.99 ± 6.64	14.61 ± 6.65	16.29 ± 6.44	<0.001
GEMA-Na	16.30 ± 6.09	16.09 ± 6.07	17.02 ± 6.08	<0.001
Length in waiting list (only transplanted)	73 (IQR 23–174)	73 (IQR 22–169)	77 (IQR 25–186)	0.363
Primary outcome	286 (4.7%)	212 (4.5%)	74 (5.4%)	0.180
Transplanted	5187 (85.5%)	4037 (85.9%)	1150 (83.7%)	0.037
Transplanted <90 days	2890 (47.6%)	2265 (48.2%)	625 (45.5%)	0.074

GEMA-Na, Gender-Equity model for liver allocation corrected by serum sodium; HCC, hepatocellular carcinoma; LT, liver transplantation; MASH, metabolic-associated steatohepatitis; MELD, Model for end-stage liver disease; MELD-Na, Model for end-stage liver disease corrected by serum sodium; MELD 3.0, Model for end-stage liver disease3.0; RFH-GFR, Royal Free Glomerular Filtration Rate.

Mortality or delisting for clinical deterioration occurred in 286 patients (4.7%) at 90 days, and in 694 patients (11.4%) as the final event on the waiting list (Fig. 1). Although waiting list time was broadly similar between men and women in the overall cohort (77 vs. 73 days; p = 0.363), women were less likely to receive a transplant (83.7% vs. 85.9%; p = 0.037). The probability of transplantation at 90 days was 48.2% in men and 45.5% in women (p = 0.074). The risk of mortality or delisting for sickness at 90 days was 4.5% in men and 5.4% in women (p = 0.180). In patients with indications other than tumours (n = 3606), women had to wait longer to receive a liver graft (70 days [IQR 21–179] vs. 58 days [IQR 17–151]; p = 0.026), and they had reduced transplantation rates at 90 days compared to men (47.6% vs. 52.3%; p = 0.012) (Supplementary Table S2).

### Predictors of mortality or delisting due to clinical deterioration

The multivariate Cox regression analysis to identify independent predictors of the primary outcome is shown in Table 2. Compared to men, women had increased risk of mortality or delisting for sickness at 90 days after controlling for clinical and analytical features (RR = 1.57 [95% confidence interval 95% CI 1.09–2.28]; p = 0.017), whereas height showed no association (RR = 1.02 [95% CI 1.00–1.03]; p = 0.067). Patients experiencing the primary outcome were also characterized by older age (RR = 1.11; p = 0.029), increased prevalence of cryptogenic cirrhosis (RR = 1.67; p = 0.037), presence of ascites despite use of diuretics (RR = 1.43; p = 0.018), increased bilirubin (RR = 1.08; p < 0.001), increased urea (RR = 1.01; p < 0.001), higher INR (RR = 1.40; p < 0.001), and lower serum sodium (RR = 0.97; p = 0.007). The combination of predictive scores and clinical characteristics revealed that female sex behaved as an independent risk factor of mortality or delisting for sickness at 90 days when using MELD (RR = 1.54 [95% CI 1.07–2.22]; p = 0.021) or MELD-Na (RR = 1.48 [95% CI 1.03–2.13]; p = 0.035). However, sex assigned at birth lost its significance in combination with GEMA-Na (RR = 1.31 [95% CI 0.91–1.87]; p = 0.149) or MELD 3.0 (RR = 1.27 [95% CI 0.88–1.82]; p = 0.204), meaning that these models successfully corrected sex-based disparities for accessing LT (Supplementary Tables S3–S6). Moderate-severe ascites increased the risk of mortality or delisting for sickness when using MELD and MELD 3.0 (p = 0.002 and p = 0.031, respectively), but it was marginally not significant when using MELD-Na (p = 0.068), and clearly lost it impact when using GEMA-Na p = 0.691) (Supplementary Tables S3–S6).

**Table 2:** Multivariate Cox's regression analysis of clinical and analytical predictors determined at inclusion in the waiting list to predict mortality or delisting for sickness at 90 days in the overall cohort (n = 6071).

Variable	β coefficient	RR	95% CI	p
Age	0.018	1.108	1.002–1.034	0.029
Sex (women)	0.452	1.572	1.085–2.277	0.017
Height (cm)	0.016	1.016	0.999–1.034	0.067
Alcohol-related liver disease	0.227	1.254	0.953–1.651	0.106
Cryptogenic cirrhosis	0.512	1.669	1.030–2.703	0.037
HCC	0.286	1.331	0.981–1.807	0.067
Diuretics use	−0.142	0.868	0.661–1.139	0.307
Ascites (moderate-severe)	0.354	1.425	1.062–1.911	0.018
Serum urea (mg/dL)	0.013	1.013	1.009–1.017	<0.001
Serum creatinine (mg/dL)	−0.207	0.813	0.621–1.063	0.131
Serum sodium (mmol/L)	−0.030	0.970	0.949–0.992	0.007
International normalized ratio	0.335	1.398	1.130–1.730	0.002
Serum albumin (g/dL)	−0.109	0.897	0.740–1.087	0.268
Serum bilirubin (mg/dL)	0.072	1.075	1.060–1.090	<0.001

95% CI, 95% confidence interval; HCC, hepatocellular carcinoma.

### Discrimination and calibration of predictive models

Table 3 shows the performance of the different scores in terms of discrimination to predict mortality or delisting for sickness within the first 90 days. In the overall cohort, the best discrimination was obtained by GEMA-Na (Hc = 0.716 [95% CI 0.682–0.750], reference), followed by MELD 3.0 (Hc = 0.706 [0.671–0.740]; p = 0.081), MELD-Na (Hc = 0.704 [95% CI 0.670–0.738]; p = 0.032), and MELD (Hc = 0.695 [95% CI 0.660–0.729]; p = 0.010). These results were similar in women although without statistical significance, maybe owing to reduced sample size. The advantage of GEMA-Na over other models was more pronounced in shorter patients (ie. height <160 cm), patients included on the waiting list due to hepatic insufficiency, and in patients with decompensated cirrhosis (Table 3). In the subcohort of patients included on the waiting list for reasons other than hepatic tumours (n = 3606; 59.4%), GEMA-Na had Hc = 0.753 (95% CI 0.715–0.792), which was significantly higher than MELD 3.0 (Hc = 0.726 [95% CI 0.686–0.767]; p = 0.001), MELD-Na (Hc = 0.726 [95% CI 0.686–0.767]; p < 0.001), and MELD (Hc = 0.703 [95% CI 0.661–0.745]; p < 0.001). The Brier scores were consistent with the above referred information, showing GEMA-Na the lowest values in the overall cohort and in the subgroups of interest (meaning best accuracy), followed by the remaining sodium-containing models (with marginal or no differences between MELD-Na and MELD 3.0), and with MELD showing the highest values (meaning the lowest accuracy) (Supplementary Table S7). Calibration analyses of GEMA-Na, MELD 3.0, MELD-Na and MELD are shown in the appendix.

**Table 3:** Harrells’ c statistics and 95% confidence intervals (in brackets) for each model in the overall cohort and in the pre-specified subgroups of interest to predict the primary outcome of the study.

Cohort	n	MELD	MELD-Na	MELD 3.0	GEMA-Na	p
Overall cohort	6071	0.695 (0.660–0.729)	0.704 (0.670–0.738)	0.706 (0.671–0.740)	0.716 (0.682–0.750)	∗p = 0.010 ∗∗p = 0.032 ∗∗∗p = 0.081
Women	1374	0.730 (0.669–0.791)	0.742 (0.680–0.804)	0.745 (0.685–0.804)	0.755 (0.694–0.816)	∗p = 0.226 ∗∗p = 0.245 ∗∗∗p = 0.436
Height <160 cm	1109	0.729 (0.656–0.803)	0.751 (0.674–0.828)	0.749 (0.673–0.825)	0.776 (0.703–0.850)	∗p = 0.009 ∗∗p = 0.024 ∗∗∗p = 0.048
Ascites (any grade)	3099	0.703 (0.661–0.745)	0.715 (0.673–0.757)	0.720 (0.679–0.760)	0.736 (0.696–0.777)	∗p = 0.013 ∗∗p = 0.001 ∗∗∗p = 0.051
Hepatic insufficiency alone	1832	0.718 (0.665–0.771)	0.751 (0.701–0.800)	0.748 (0.698–0.798)	0.771 (0.723–0.819)	∗p = 0.004 ∗∗p = 0.039 ∗∗∗p = 0.049
Decompensated cirrhosis^a^	2887	0.700 (0.653–0.747)	0.728 (0.683–0.773)	0.725 (0.680–0.771)	0.753 (0.711–0.796)	∗p < 0.001 ∗∗p = 0.002 ∗∗∗p = 0.004
Non-tumoral indications^b^	3606	0.703 (0.661–0.745)	0.726 (0.686–0.767)	0.726 (0.686–0.767)	0.753 (0.715–0.792)	∗p < 0.001 ∗∗p < 0.001 ∗∗∗p = 0.001

*p* values for comparing discrimination are shown for GEMA-Na vs. MELD (∗), for GEMA-Na vs. MELD-Na (∗∗) and for GEMA-Na vs. MELD 3.0 (∗∗∗) and were obtained from the statistical test described by Kang et al.^16^

a Comprised hepatic insufficiency, refractory ascites, and recurrent/persistent hepatic encephalopathy.

b After excluding hepatocellular carcinoma and other primary or secondary liver tumors.

### Reclassification and differential prioritization

**Fig. 2:**
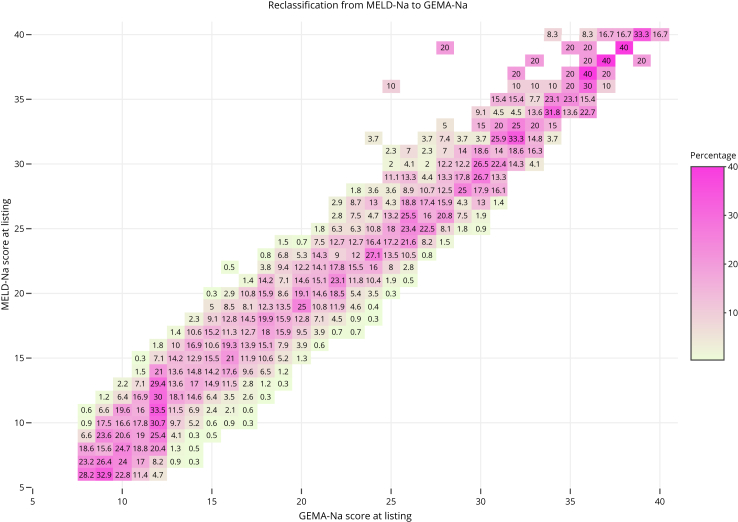
Reclassification diagram between the Gender-Equity Model for liver Allocation corrected by serum sodium (GEMA-Na) and the Model for End Stage Liver Disease corrected by serum sodium (MELD-Na) in the whole study population (n = 6071). The diagonal line shows coinciding values of compared models. For a given MELD-Na score, deviations to the left indicate lower GEMA-Na and deviations to the right indicate higher GEMA-Na. Numbers in each box indicate percentages of change.

**Fig. 3:**
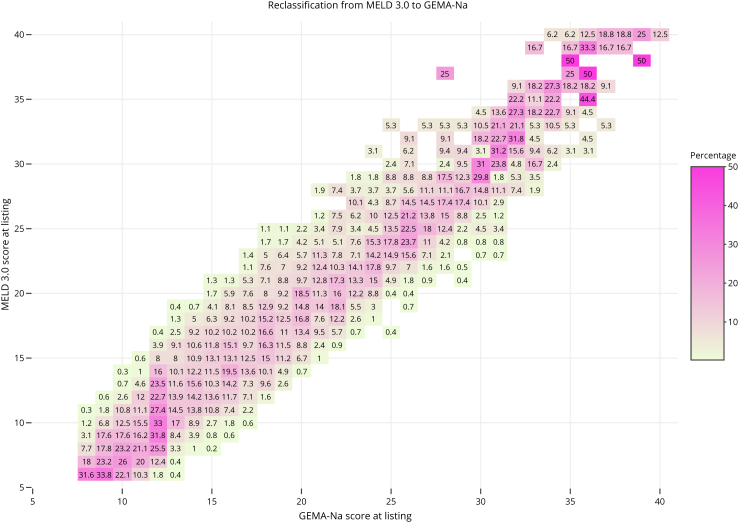
Reclassification diagram between the Gender-Equity Model for liver Allocation corrected by serum sodium (GEMA-Na) and the Model for End Stage Liver Disease 3.0 (MELD 3.0) in the whole study population (n = 6071). The diagonal line shows coinciding values of compared models. For a given MELD 3.0 score, deviations to the left indicate lower GEMA-Na and deviations to the right indicate higher GEMA-Na. Numbers in each box indicate percentages of change.

Reclassification and differential prioritization analyses were assessed in sodium-containing models (ie. MELD-Na, MELD 3.0, and GEMA-Na). Figs. 2 and 3 show the reclassification diagrams between GEMA-Na and MELD-Na, and GEMA-Na and MELD 3.0, respectively. The transition from MELD-Na to GEMA-Na would change the score by 2 or more points in 3170 patients (52.3%), 36.8% upgraded and 15.5% downgraded. When comparing MELD 3.0 vs. GEMA-Na, 3899 patients (64.2%) would change the score by 2 or more points, 51.4% upgraded and 12.8% downgraded.

**Fig. 4:**
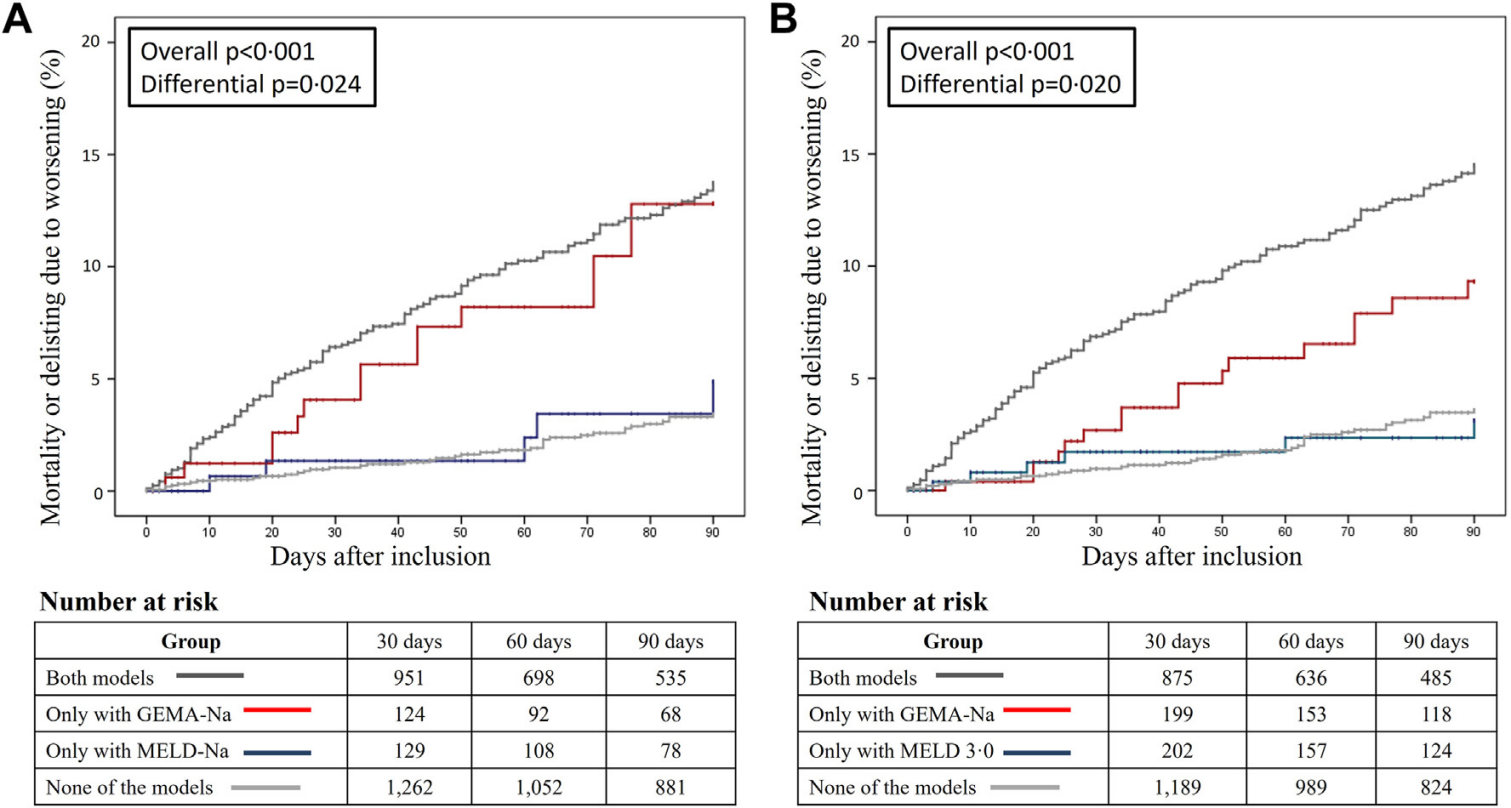
Cumulative incidence of mortality or delisting due to clinical deterioration with right-censoring at day 90 after listing. Patients were stratified according to the differential prioritization between GEMA-Na and MELD-Na (panel A), and between GEMA-Na and MELD 3.0 (panel B) as follows: both models agreed to transplant (dark grey curve), only GEMA-Na prioritized for transplantation (red curve), only the comparator prioritized for transplantation (blue curve), and none of the models assigned prioritization for transplantation (light grey). p values correspond to log rank test considering the four strata (overall p), or only considering differentially prioritized patients (differential p). GEMA-Na: Gender-Equity Model for liver Allocation corrected by serum sodium; MELD-Na: Model for End stage Liver Disease corrected by serum sodium; MELD 3.0: Model for End stage Liver Disease 3.0.

Differential prioritization was assessed in patients with indications other than tumours (n = 3606) in whom urgency-based scores are usually implemented. Among 1838 LT procedures performed within the first 90 days, differential prioritization occurred in 168 patients (9.1%) between MELD-Na and GEMA-Na, and in 270 patients (14.7%) between MELD 3.0 and GEMA-Na (Supplementary Tables S8 and S9, respectively). Patients differentially prioritized by GEMA-Na had increased risk of mortality or delisting for sickness at 90 days compared to patients differentially prioritized by MELD-Na (8.9% vs. 3%; p = 0.021), or by MELD 3.0 (6.3% vs 2.2%; p = 0.019). Fig. 4 shows that the cumulative incidence of mortality or delisting for sickness within the first 90 days was higher in patients differentially prioritized by GEMA-Na, compared to that observed in the groups differentially prioritized by MELD-Na (log rank p = 0.024) and MELD 3.0 (log rank p = 0.020). Outcomes of patients differentially prioritized by MELD-Na and MELD 3.0 mirrored those of patients in which all models agreed to assign low priority. Within the first 90 days, the implementation of GEMA-Na would avoid one in 20 deaths overall compared with MELD-Na (one in 15 deaths in women), and one in 18 deaths overall compared with MELD 3.0 (one in 30 deaths in women).

GEMA-Na prioritized more women (47.6% vs. 20%), more patients with moderate-severe ascites (72.6% vs. 29.8%), and patients with reduced height (163.99 ± 8.64 cm vs. 169.35 ± 8.74 cm) than MELD-Na (p < 0.001 for all comparisons). MELD 3.0 prioritized more women than GEMA-Na (33.3% vs. 25.6%; p = 0.047) but a reduced proportion of patients with moderate-severe ascites (29.6% vs. 79.3%; p < 0.001). Noteworthy, GEMA-Na differentially prioritized a group of patients with worse renal function but relatively lower INR and serum bilirubin (Supplementary Tables S8 and S9).

## Discussion

In this nationwide cohort study, we confirmed sex-based disparities for accessing LT in a context of high availability of donors and we compared novel scores specifically designed to address this inequity. Both MELD 3.0 and GEMA-Na were able to correct sex inequities for accessing LT. However, GEMA-Na outperformed MELD-Na and MELD 3.0 in terms of discrimination and the transition to GEMA-Na would save a clinically relevant number of lives.

Sex-disparities for accessing LT became evident after the implementation of MELD in the United States.^3^ Women had 30% increased risk of mortality or delisting for sickness compared to men despite relevant MELD updates.^18^ It has been estimated that 800 women's deaths could have been avoided over the last decade in the United States if women had equal access to deceased donor LT as men.^5^ The information about sex disparities in other countries is scarce but it is deemed relevant as local environmental factors such as allocation policies, donor availability, length of the waiting list, and indications for LT could influence the access to LT. Spain has the highest deceased donation rates worldwide and the waiting list for LT has been shortened over the last few years.^12^ In addition, waitlist mortality rates are consistently below 5% and allocation policies are heterogenous among centres. Despite this challenging scenario, we found that women had 57% excess risk of mortality or delisting for clinical deterioration compared to men after controlling for clinical features and analytical parameters. In addition, among patients included on the waiting list for indications other than tumours, the length on the waiting list was 17% longer for women and their likelihood of transplantation at 90 days was reduced. These figures mirror those reported in the United States.^4^

Sex disparities for accessing LT are likely multifactorial.^6^ Men and women show different indications for LT which could justify asymmetrical allocation priorities. In addition, women are shorter in average than men and may have reduced abdominal capacity.^19^ In the MELD 3.0 study, both height and sex were tested as covariates in the model and the effect of sex was larger and more consistent than that of height.^8^ Another analysis of the Organ Procurement and Transplantation Network database demonstrated that even the tallest women (>170 cm) were 10% less likely to receive a LT than men of the same height.^6^ Our results align with these observations. Height and sex were included in the multivariate analysis to predict the primary outcome, and only female sex was statistically significant, meaning a diminished impact of height, which could act as a confounder. The most relevant factor determining sex inequities is serum creatinine within the MELD score.^6^ Renal impairment is an undoubtful predictor of mortality in patients with end-stage liver disease, but it is strongly influenced by muscle mass, which is in average greater in men than in women.^20^ With identical glomerular filtration rate, women could receive up to 4.9 less creatinine-derived MELD points, thus justifying their reduced priority.^6^

MELD 3.0 and GEMA-Na corrected sex-based disparities for accessing LT in this nationwide cohort, but the approach of both models differed. MELD 3.0 included sex in the model and assigned 1.3 extra points to women which equals to the average score gap between men and women in the United States. However, sex-based disparities are not homogeneous so that the gap of creatinine-derived MELD points between men and women ranges between 0.5 and 4.9, depending on the renal function.^6^ Therefore, MELD 3.0 may overestimate or underestimate the risk of waitlist mortality in women depending on their glomerular filtration rate, and its expected performance would be worse outside the United States where the extent of sex disparities may be different. Indeed, MELD 3.0 failed to demonstrate a significant discrimination improvement than MELD-Na in South Korea,^20^ Italy,^11^ United Kingdom,^9^ Australia,^9^ and in the present study. We found that MELD 3.0 prioritized more women than GEMA-Na but their likelihood of death or delisting for sickness was lower, meaning an inappropriate compensation. On the other hand, GEMA-Na replaced serum creatinine by the RFH-GFR which resulted in a complete removal of creatinine-derived bias. RFH-GFR has been developed and externally validated in individuals with chronic liver disease, including decompensated cirrhosis.^10^ RFH-GFR has been criticized because it incorporates moderate-severe ascites in the equation, being this factor a potential source of subjectivity. We have proposed an objective evaluation of moderate-severe ascites defined as either the need of a large volume paracentesis within the previous 4 weeks, or a compatible physical examination further confirmed with an abdominal ultrasound or computed tomography, which are readily available techniques routinely performed in LT candidates. Under these conditions, the vulnerability of ascites to bias would be diminished. The differential prioritization analysis between MELD 3.0 and GEMA-Na revealed that GEMA-Na better captured the interplay between ascites, renal impairment, and hyponatremia to assign extra-prioritization to patients historically disadvantaged by the MELD family scores. An ensemble model combining GEMA-Na and MELD 3.0 would provide limited benefit since the group of patients differentially prioritized by MELD 3.0 had identical outcomes as the group in which both models agreed not to transplant. In addition, an ensemble model would make it harder to understand how the score is derived in a particular patient, which would be a barrier for its implementation in clinical practice. GEMA-Na is the only model able to amend sex disparities for accessing LT which has been externally validated, consistently showing better discrimination than MELD-Na (and MELD 3.0) in Australia,^9^ Italy,^11^ and also in Spain according to our results.

The present study is limited by the retrospective collection of some analytical values not available in the ONT database. In addition, the high availability of donors in Spain may have resulted into a relatively reduced number of primary outcome events. Therefore, the statistical power of some sensitivity analyses in subgroups of interest, including women, could have been compromised, thus claiming for caution when interpreting not significant trends. Analyses according to the aetiology of liver disease could not be performed as there would be an insufficient number of events to allow meaningful comparisons. The Brier score is known for its limited generalization capacity and its results should be interpreted alongside other discrimination metrics provided in the manuscript. Finally, our decision to account for all LT candidates, including patients with hepatic tumours, could have impacted negatively on the discrimination and calibration of all predictive models, but in turn provided with more realistic data of the actual composition of the waiting list and the potential impact of transitioning from one model to another.

In conclusion, there are meaningful sex disparities for accessing LT in Spain which rest on biased estimations of renal function among patients with end-stage liver disease. Although both MELD 3.0 and GEMA-Na could amend these inequities, only GEMA-Na produced more accurate predictions of waiting list outcomes, aligning with findings from other geographic areas with different allocation policies. Unless future evidence proves otherwise, GEMA-Na could be considered the standard-of-care for LT waiting list prioritization.

## Contributors

MLR-P: study conception and design, obtained funding, data analysis, drafting the manuscript, and study guarantor.

GdR and CHM: data curation, analysis, and critical revision of the article.

AMG-O: mathematical modelling, data analysis and critical revision of the article.

MVA, TPV, SP, MLO, GP, FS, RGG, AC, ST, MB, RMM, SP, MR, IB, CAM, EO, LGD, MDE, AAM, GBF, SL, ACL, ARG, CSC, CC, JAP, JC, AO, AAN, SRM, MRS: acquisition of data and critical revision of the article.

DG-R: data curation and analysis.

MG: data curation, analysis, and critical revision of the article.

MLR-P and AMG-O had full access to the study underlying data and verified results. All authors revised and approved the final version of the article and had final responsibility for the decision to submit for publication.

## Data sharing statement

The data used the study was extracted from the Organización Nacional de Trasplantes (ONT). Deidentified participant data could be shared with an external investigator only after approval by ONT. For this purpose, proposals must be referred to the representative of ONT in the present study, Mrs. Gloria De la Rosa, by e-mail at grosa@sanidad.gob.es. A signed confidentiality agreement would be required.

## Declaration of interests

MLR-P has received lecture fees from Chiesi and Advanz Pharma, outside the present work. RM-M has received lecture fees from Chiesi, outside the present work. JC has received lecture fees from Chiesi and Astellas, outside the present work. All other authors declare no competing interests.

## Figures and Tables

**Supplementary Fig. S1:**
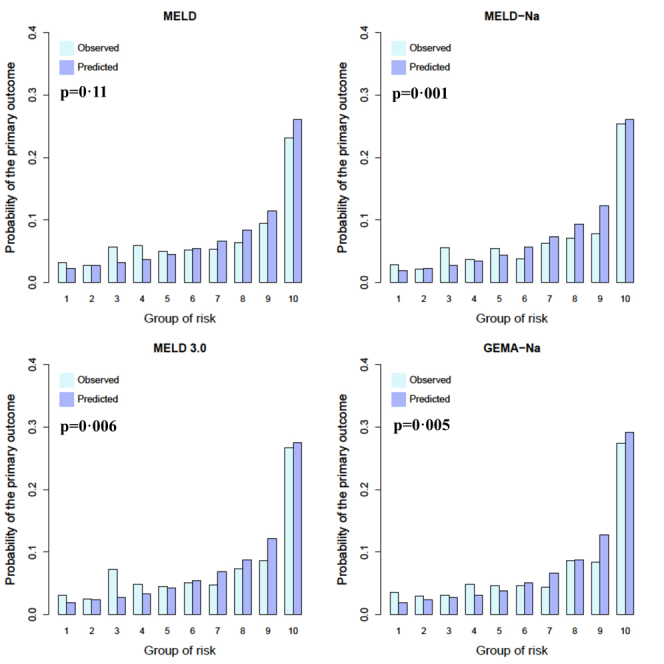


**Supplementary Fig. S2:**