# Supplementary material for: GEMA-Na and MELD 3.0 severity scores to address sex disparities for accessing liver transplantation: a nationwide retrospective cohort study
Source: eClinicalMedicine. 2024 Jul 18;74:102737. doi: 10.1016/j.eclinm.2024.102737 (PMC11304699; doi:10.1016/j.eclinm.2024.102737)
Supplement: Supplementary materials [file mmc1.docx]

**APPENDIX**

**“Transparent Reporting of a multivariable prediction model for Individual Prognosis Or Diagnosis (TRIPOD)” explanation and elaboration checklist.**

**A nationwide cohort study to amend sex disparities for accessing liver transplantation.**

| **Section/Topic** | **Item** |  | | **Checklist Item** | **Page** |
| --- | --- | --- | --- | --- | --- |
| **Title and abstract** | | | | | |
| Title | 1 | | Identify the study as developing and/or validating a multivariable prediction model, the target population, and the outcome to be predicted. | | 1 |
| Abstract | 2 | | Provide a summary of objectives, study design, setting, participants, sample size, predictors, outcome, statistical analysis, results, and conclusions. | | 10 |
| **Introduction** | | | | | |
| Background and objectives | 3a | | Explain the medical context (including whether diagnostic or prognostic) and rationale for developing or validating the multivariable prediction model, including references to existing models. | | 13-14 |
|  | 3b | | Specify the objectives, including whether the study describes the development or validation of the model or both. | | 14 |
| **Methods** | | | | | |
| Source of data | 4a | | Describe the study design or source of data (e.g., randomized trial, cohort, or registry data), separately for the development and validation data sets, if applicable. | | 14-15 |
|  | 4b | | Specify the key study dates, including start of accrual; end of accrual; and, if applicable, end of follow-up. | | 14 |
| Participants | 5a | | Specify key elements of the study setting (e.g., primary care, secondary care, general population) including number and location of centres. | | 14-15 |
|  | 5b | | Describe eligibility criteria for participants. | | 14 |
|  | 5c | | Give details of treatments received, if relevant. | | N/A |
| Outcome | 6a | | Clearly define the outcome that is predicted by the prediction model, including how and when assessed. | | 16 |
|  | 6b | | Report any actions to blind assessment of the outcome to be predicted. | | N/A |
| Predictors | 7a | | Clearly define all predictors used in developing or validating the multivariable prediction model, including how and when they were measured. | | 15-16 |
|  | 7b | | Report any actions to blind assessment of predictors for the outcome and other predictors. | | N/A |
| Sample size | 8 | | Explain how the study size was arrived at. | | * |
| Missing data | 9 | | Describe how missing data were handled (e.g., complete-case analysis, single imputation, multiple imputation) with details of any imputation method. | | 15 |
| Statistical analysis methods | 10a | | Describe how predictors were handled in the analyses. | | * |
|  | 10b | | Specify type of model, all model-building procedures (including any predictor selection), and method for internal validation. | | * |
|  | 10c | | For validation, describe how the predictions were calculated. | | N/A |
|  | 10d | | Specify all measures used to assess model performance and, if relevant, to compare multiple models. | | 16-17 |
|  | 10e | | Describe any model updating (e.g., recalibration) arising from the validation, if done. | | N/A |
| Risk groups | 11 | | Provide details on how risk groups were created, if done. | | 16 |
| Development vs. validation | 12 | | For validation, identify any differences from the development data in setting, eligibility criteria, outcome, and predictors. | | N/A |
| **Results** | | | | | |
| Participants | 13a | | Describe the flow of participants through the study, including the number of participants with and without the outcome and, if applicable, a summary of the follow-up time. A diagram may be helpful. | | 17 |
|  | 13b | | Describe the characteristics of the participants (basic demographics, clinical features, available predictors), including the number of participants with missing data for predictors and outcome. | | 17-18 |
|  | 13c | | For validation, show a comparison with the development data of the distribution of important variables (demographics, predictors and outcome). | | N/A |
| Model development | 14a | | Specify the number of participants and outcome events in each analysis. | | 18 |
|  | 14b | | If done, report the unadjusted association between each candidate predictor and outcome. | | 18 |
| Model specification | 15a | | Present the full prediction model to allow predictions for individuals (i.e., all regression coefficients, and model intercept or baseline survival at a given time point). | | * |
|  | 15b | | Explain how to the use the prediction model. | | 18 |
| Model performance | 16 | | Report performance measures (with CIs) for the prediction model. | | 19-21 |
| Model-updating | 17 | | If done, report the results from any model updating (i.e., model specification, model performance). | | N/A |
| **Discussion** | | | | | |
| Limitations | 18 | | Discuss any limitations of the study (such as nonrepresentative sample, few events per predictor, missing data). | | 24 |
| Interpretation | 19a | | For validation, discuss the results with reference to performance in the development data, and any other validation data. | | 23-24 |
|  | 19b | | Give an overall interpretation of the results, considering objectives, limitations, results from similar studies, and other relevant evidence. | | 21-23 |
| Implications | 20 | | Discuss the potential clinical use of the model and implications for future research. | | 24-25 |
| **Other information** | | | | | |
| Supplementary information | 21 | | Provide information about the availability of supplementary resources, such as study protocol, Web calculator, and data sets. | | * |
| Funding | 22 | | Give the source of funding and the role of the funders for the present study. | | 32-33 |

* A detailed supplementary material file is provided.

**Calculation of predicting scores.**

Predicting scores were calculated at the inclusion in the waiting list. Serum bilirubin, international normalized ratio (INR), and serum creatinine were required to calculate MELD, with the addition of serum sodium for MELD-Na according to established formulas^1^. Serum albumin and sex assigned at birth were considered specifically to calculate MELD 3·0 (online calculator at https://medcalculators.stanford.edu/meld)^2^. Finally, to calculate GEMA-Na, we combined serum bilirubin, INR, serum sodium, and the RFH-GFR^3^, which in turn comprised age, sex assigned at birth, serum urea, serum creatinine, INR, serum sodium, and moderate-severe ascites as a binary variable^4^. As some institutions had blood urea nitrogen (BUN) available instead of serum urea, conversions were made as follows: Urea (mmol/l) = BUN (mg/dl) * 0.357. An online calculator for GEMA-Na is available at: https://en.gemascore.com/.

**Methodology for sample size calculation.**

We estimated the minimum sample size required to compare the discrimination between the Gender-Equity Model for Liver Allocation corrected by serum sodium (GEMA-Na) and the Model for End Stage Liver Disease 3·0 (MELD 3·0) to predict mortality or delisting for sickness within the first 90 days from listing. We followed the method proposed by Jinks et al. which was specifically designed for multivariate prognostic models for time-to-event data considering right-censoring^5^. The authors considered for their formulae the Royston & Sauerbrei D measure of discrimination^6^, which could be derived from the Harrell c-index. The expected results on discrimination of GEMA-Na and MELD 3·0, and the prevalence of the primary outcome were obtained from the external validation cohort of the study by Rodríguez-Perálvarez et al.^3^. The following assumptions were made:

- Expected D statistic of GEMA-Na: 1·9244658, standard error (D) 0·2044043
- Expected D statistic of MELD 3·0: 1·79807867, standard error (D) 0·20447317
- Expected proportion of patients experiencing the primary outcome: 5·5%
- Statistical power: 90%
- Proportion of censoring: 50%
- Alpha error: 0·05.

Under these premises the minimum sample size would be 4,932 patients, including 271 patients experiencing the primary outcome. The present study included 6,071 patients with 286 patients experiencing the primary outcome.

**Calibration of predicting models.**

In the whole study population, MELD was adequately calibrated (χ2=14·494; p=0·106) but those models including serum sodium showed poorer calibration: MELD-Na χ2=26·955 (p=0·001), MELD 3·0 χ2=23·250 (p=0·006), and GEMA-Na χ2=23·473 (p=0·005) (supplementary figure 1). In the analysis of patients with indications other than tumours, all models were adequately calibrated: MELD χ2=9·310 (p=0·409), MELD-Na χ2=11·700 (p=0·231), MELD 3·0 χ2=11·209 (p=0·262), and GEMA-Na χ2=10·229 (p=0·332) (supplementary figure 2).

**Supplementary table 1.** Distribution of the study population according to the hospital in which patients were enlisted for liver transplantation.

| **HOSPITAL** | **AUTONOMOUS REGION** | **n** |
| --- | --- | --- |
| La Fe | Comunidad Valenciana | 565 |
| Cruces | Basque Country | 431 |
| Virgen del Rocío | Andalusia | 400 |
| Hospital Clínic | Catalonia | 368 |
| Bellvitge | Catalonia | 341 |
| A Coruña | Galicia | 337 |
| Virgen de la Arrixaca | Murcia | 316 |
| Reina Sofía | Andalusia | 292 |
| Santiago | Galicia | 292 |
| Ramón y Cajal | Madrid | 283 |
| Doce de Octubre | Madrid | 270 |
| Alicante | Comunidad Valenciana | 245 |
| Vall D’Hebron | Catalonia | 237 |
| Rio Hortega | Castilla y León | 215 |
| Nuestra Sra de la Candelaria | Canary Islands | 221 |
| Central de Asturias | Asturias | 200 |
| Puerta de Hierro | Madrid | 177 |
| Virgen de las Nieves | Andalusia | 173 |
| Badajoz | Extremadura | 163 |
| Regional de Málaga | Andalusia | 154 |
| Lozano Blesa | Aragón | 142 |
| Navarra | Comunidad foral de Navarra | 108 |
| Marqués de Valdecilla | Santander | 98 |
| Gregorio Marañón | Madrid | 43 |
| Overall |  | 6,071 |

**Supplementary table 2.** Clinical features of 3,606 patients enlisted for a first deceased donor liver transplantation for indications other than tumours in Spain from 2016 to 2021.

| **VARIABLE** | **OVERALL**  **(N=3,606)** | **MEN**  **(N=2,598)** | **WOMEN**  **(N=1,008)** | **P** |
| --- | --- | --- | --- | --- |
| Age | 56·62 ± 9·34 | 57·10 ± 8·44 | 55·38 ± 11·26 | <0·001 |
| Height (cm) | 168·05 ± 8·91 | 171·39 ± 7·15 | 159·45 ± 6·99 | <0·001 |
| Weight (kg) | 76·57 ± 16·11 | 80·75 ± 14·95 | 65·80 ± 13·82 | <0·001 |
| Body mass index (kg/m2) | 27·06 ± 4·98 | 27·50 ± 4·78 | 25·93 ± 5·28 | <0·001 |
| Aetiology (alcohol) | 2,046 (56·7%) | 1,759 (67·7%) | 287 (28·5%) | <0·001 |
| Aetiology (hepatitis C) | 489 (13·6%) | 383 (14·7%) | 106 (10·5%) | 0·001 |
| Aetiology (hepatitis B) | 160 (4·4%) | 137 (5·3%) | 23 (2·3%) | <0·001 |
| Aetiology (autoimmune) | 449 (12·5%) | 156 (6%) | 293 (29·1%) | <0·001 |
| Aetiology (MASH) | 71 (2%) | 42 (1·6%) | 29 (2·9%) | 0·014 |
| Aetiology (cryptogenic) | 198 (5·5%) | 110 (4·2%) | 88 (8·7%) | <0·001 |
| Aetiology (others) | 444 (12·3%) | 227 (8·7%) | 217 (21·5%) | <0·001 |
| Use of diuretics at inclusion | 2,391 (66·5%) | 1,758 (68%) | 633 (62·9%) | 0·004 |
| Ascites  No  Mild  Moderate-severe | 1,028 (28·5%)  648 (18%)  1,930 (53·5%) | 669 (25·8%)  463 (17·8%)  1,466 (56·4%) | 359 (36·6%)  185 (18·4%)  464 (46%) | <0·001 |
| Urea (mg/dL) | 45·44 ± 29·80 | 46·54 ± 30·87 | 42·63 ± 26·71 | <0·001 |
| Creatinine (mg/dL) | 0·99 ± 0·59 | 1·03 ± 0·58 | 0·88 ± 0·58 | <0·001 |
| RFH-GFR (ml/min) | 63·66 ± 25·83 | 63·97 ± 25·67 | 62·85 ± 26·23 | 0·247 |
| International Normalized Ratio | 1·56 ± 0·52 | 1·59 ± 0·52 | 1·49 ± 0·49 | <0·001 |
| Bilirubin (mg/dL) | 2·80 (IQR 1·50-5·20) | 2·80 (IQR 1·60-5·10) | 2·70 (IQR 1·40-5·50) | 0·693 |
| Sodium (mmol/L) | 136·16 ± 5·04 | 135·94 ± 5·07 | 136·73 ± 4·91 | <0·001 |
| Albumin (g/dL) | 3·24 ± 0·63 | 3·23 ± 0·62 | 3·27 ± 0·66 | 0·115 |
| MELD | 16·22 ± 5·74 | 16·57 ± 5·74 | 15·33 ± 5·63 | 0·368 |
| MELD-Na | 18·64 ± 6·13 | 19·06 ± 6·07 | 17·55 ± 6·14 | 0·290 |
| MELD 3·0 | 18·14 ± 6·23 | 18·15 ± 6·23 | 18·11 ± 6·23 | 0·855 |
| GEMA-Na | 19·07 ± 5·90 | 19·23 ± 5·88 | 18·67 ± 5·95 | 0·010 |
| Length in waiting list (only transplanted) | 60 (IQR 18-158) | 58 (IQR 17-151) | 70 (IQR 21-179) | 0·026 |
| Primary outcome | 201 (5·6%) | 141 (5·4%) | 60 (6%) | 0·537 |
| Transplanted | 3,026 (83·9%) | 2,188 (84·2%) | 838 (83·1%) | 0·427 |
| Transplanted <90 days | 1,838 (51%) | 1,358 (52·3%) | 480 (47·6%) | 0·012 |

GEMA-Na: Gender-Equity Model for liver Allocation corrected by serum sodium; HCC: hepatocellular carcinoma; LT: liver transplantation; MASH: metabolic-associated steatohepatitis; MELD: Model for End-stage Liver Disease; MELD-Na: Model for End-stage Liver Disease corrected by serum sodium; MELD 3.0: Model for end-stage liver disease 3.0; RFH-GFR: Royal Free Hospital Glomerular Filtration Rate.

**Supplementary table 3**. Multivariate Cox’s regression analysis of clinical features combined with the Model for End-stage Liver Disease (MELD) determined at inclusion in the waiting list to predict mortality or delisting for sickness at 90 days in the overall cohort (n=6,071).

| **Variable** | **β coefficient** | **RR** | **95%CI** | **p** |
| --- | --- | --- | --- | --- |
| Age | 0·024 | 1·024 | 1·008-1·041 | 0·003 |
| Sex (women) | 0·431 | 1·539 | 1·067-2·219 | 0·021 |
| Height (cm) | 0·011 | 1·011 | 0·994-1·029 | 0·210 |
| Alcohol-related liver disease | 0·116 | 1·123 | 0·857-1·473 | 0·400 |
| Cryptogenic cirrhosis | 0·438 | 1·550 | 0·956-2·515 | 0·076 |
| HCC | 0·337 | 1·401 | 1·036-1·895 | 0·029 |
| Diuretics use | -0·293 | 0·746 | 0·574-0·969 | 0·028 |
| Ascites (moderate-severe) | 0·452 | 1·571 | 1·186-2·080 | 0·002 |
| MELD | 0·135 | 1·144 | 1·122-1·167 | <0·001 |

95%CI: 95% confidence interval; HCC: hepatocellular carcinoma.

**Supplementary table 4**. Multivariate Cox’s regression analysis of clinical features combined with the Model for End-stage Liver Disease corrected with serum sodium (MELD-Na) determined at inclusion in the waiting list to predict mortality or delisting for sickness at 90 days in the overall cohort (n=6,071).

| **Variable** | **β coefficient** | **RR** | **95%CI** | **p** |
| --- | --- | --- | --- | --- |
| Age | 0·023 | 1·023 | 1·007-1·039 | 0·005 |
| Sex (women) | 0·392 | 1·480 | 1·029-2·129 | 0·035 |
| Height (cm) | 0·011 | 1·011 | 0·994-1·028 | 0·224 |
| Alcohol-related liver disease | 0·073 | 1·075 | 0·821-1·409 | 0·598 |
| Cryptogenic cirrhosis | 0·430 | 1·537 | 0·948-2·492 | 0·081 |
| HCC | 0·369 | 1·447 | 1·070-1·956 | 0·016 |
| Diuretics use | -0·312 | 0·732 | 0·565-0·948 | 0·018 |
| Ascites (moderate-severe) | 0·263 | 1·301 | 0·981-1·725 | 0·068 |
| MELD-Na | 0·134 | 1·144 | 1·121-1·166 | <0·001 |

95%CI: 95% confidence interval; HCC: hepatocellular carcinoma.

**Supplementary table 5**. Multivariate Cox’s regression analysis of clinical features combined with the Model for End-stage Liver Disease 3·0 (MELD 3·0) determined at inclusion in the waiting list to predict mortality or delisting for sickness at 90 days in the overall cohort (n=6,071).

| **Variable** | **β coefficient** | **RR** | **95%CI** | **p** |
| --- | --- | --- | --- | --- |
| Age | 0·024 | 1·025 | 1·009-1·041 | 0·002 |
| Sex (women) | 0·236 | 1·266 | 0·880-1·823 | 0·204 |
| Height (cm) | 0·012 | 1·012 | 0·994-1·029 | 0·186 |
| Alcohol-related liver disease | 0·091 | 1·095 | 0·836-1·435 | 0·508 |
| Cryptogenic cirrhosis | 0·409 | 1·506 | 0·928-2·442 | 0·097 |
| HCC | 0·407 | 1·502 | 1·109-2·035 | 0·009 |
| Diuretics use | -0·302 | 0·739 | 0·570-0·958 | 0·022 |
| Ascites (moderate-severe) | 0·310 | 1·363 | 1·029-1·806 | 0·031 |
| MELD 3·0 | 0·132 | 1·141 | 1·119-1·163 | <0·001 |

95%CI: 95% confidence interval; HCC: hepatocellular carcinoma.

**Supplementary table 6**. Multivariate Cox’s regression analysis of clinical features combined with Gender-Equity Model for liver Allocation corrected by serum sodium (GEMA-Na) determined at inclusion in the waiting list to predict mortality or delisting for sickness at 90 days in the overall cohort (n=6,071).

| **Variable** | **β coefficient** | **RR** | **95%CI** | **p** |
| --- | --- | --- | --- | --- |
| Age | 0·015 | 1·015 | 1·000-1·031 | 0·057 |
| Sex (women) | 0·266 | 1·305 | 0·909-1·873 | 0·149 |
| Height (cm) | 0·011 | 1·011 | 0·994-1·029 | 0·192 |
| Alcohol-related liver disease | 0·083 | 1·086 | 0·829-1·423 | 0·547 |
| Cryptogenic cirrhosis | 0·402 | 1·495 | 0·921-2·427 | 0·104 |
| HCC | 0·434 | 1·543 | 1·136-2·094 | 0·005 |
| Diuretics use | -0·279 | 0·757 | 0·585-0·979 | 0·034 |
| Ascites (moderate-severe) | 0·059 | 1·060 | 0·794-1·416 | 0·691 |
| GEMA-Na | 0·154 | 1·166 | 1·142-1·191 | <0·001 |

95%CI: 95% confidence interval; HCC: hepatocellular carcinoma.

**Supplementary table 7.** Brier scores obtained from each model in the overall cohort and in subgroups of interest. The lower values indicate higher accuracy.

| **COHORT** | **n** | **MELD** | **MELD-Na** | **MELD 3.0** | **GEMA-Na** |
| --- | --- | --- | --- | --- | --- |
| Overall cohort | 6,071 | 0·0524 | 0·0517 | 0·0517 | 0·0507 |
| Women | 1,374 | 0·0548 | 0·0540 | 0·0539 | 0·0528 |
| Height <160 cm | 1,109 | 0·0482 | 0·0462 | 0·0473 | 0·0454 |
| Ascites (any grade) | 3,099 | 0·0687 | 0·0673 | 0·0674 | 0·0652 |
| Hepatic insufficiency alone | 1,832 | 0·0675 | 0·0640 | 0·0650 | 0·0620 |
| Decompensated cirrhosis^1^ | 2,887 | 0·0630 | 0·0607 | 0·0613 | 0·0587 |
| Non-tumoral indications^2^ | 3,606 | 0·0600 | 0·0583 | 0·0586 | 0·0564 |

MELD: Model for End-stage Liver Disease; MELD-Na: Model for End-stage Liver Disease corrected by serum sodium; MELD 3·0: Model for end-stage liver disease 3·0; GEMA-Na: Gender-Equity Model for liver Allocation corrected by serum sodium.

^1^ Comprised hepatic insufficiency, refractory ascites, and recurrent/persistent hepatic encephalopathy.

^2^ After excluding hepatocellular carcinoma and other primary or secondary liver tumors.

**Supplementary table 8.** Clinical characteristics of patients included in the waiting list for indications other than tumours who were assigned a liver graft in the simulation analysis. Patients were stratified as follows: both models agreed to transplant, only MELD-Na prioritized for liver transplantation, or only GEMA-Na prioritized for liver transplantation. The p values denote comparisons between patients differently prioritized by MELD-Na vs. GEMA-Na.

| **VARIABLE** | **Transplanted both (n=1,670)** | **MELD-Na transplanted (N=168)** | **GEMA-Na transplanted (N=168)** | **p** |
| --- | --- | --- | --- | --- |
| Age | 57·07 ± 8·55 | 53·61 ± 9·66 | 60·77 ± 7·05 | <0·001 |
| Sex (women) | 418 (25%) | 34 (20%) | 80 (47·6%) | <0·001 |
| Height (cm) | 168·22 ± 8·81 | 169·35 ± 8·74 | 163·99 ± 8·64 | <0·001 |
| Weight (kg) | 76·93 ± 15·58 | 79·41 ± 17·25 | 73·42 ± 16·69 | 0·001 |
| Body mass index (kg/m2) | 27·16 ± 4·83 | 27·62 ± 5·22 | 27·22 ± 5·41 | 0·492 |
| Ascites (moderate-severe) | 1,104 (66·1%) | 122 (72·6%) | 50 (29·8%) | <0·001 |
| Urea (mg/dL) | 55·13 ± 36·14 | 23·44 ± 7·26 | 63·59 ± 27·98 | <0·001 |
| Creatinine (mg/dL) | 1·17 ± 0·76 | 0·62 ± 0·11 | 1·19 ± 0·34 | <0·001 |
| RFH-GFR (ml/min) | 52·17 ± 21·83 | 95·53 ± 15·78 | 42·85 ± 12·04 | <0·001 |
| International Normalized Ratio | 1·80 ± 0·63 | 1·73 ± 0·30 | 1·35 ± 0·22 | <0·001 |
| Bilirubin (mg/dL) | 4·70 (IQR 2·60-8·50) | 4·20 (IQR 3·10-5·60) | 1·70 (IQR 1·00-3·20) | <0·001 |
| Sodium (mmol/L) | 133·40 ± 5·31 | 137·23 ± 3·28 | 137·13 ± 3·69 | 0·803 |
| Albumin (g/dL) | 3·14 ± 0·64 | 3·01 ± 0·52 | 3·31 ± 0·64 | <0·001 |
| Primary outcome | 141 (8·4%) | 5 (3%%) | 15 (8·9%) | 0·021 |

RFH-GFR: Royal Free Glomerular Filtration Rate

**Supplementary table 9.** Clinical characteristics of patients included in the waiting list for indications other than tumours who were assigned a liver graft in the simulation analysis. Patients were stratified as follows: both models agreed to transplant, only MELD 3·0 prioritized for liver transplantation, or only GEMA-Na prioritized for liver transplantation. The p values denote comparisons between patients differently prioritized by MELD-Na vs. GEMA-Na.

| **VARIABLE** | **Transplanted both (n=1,568)** | **MELD 3.0 transplanted (N=270)** | **GEMA-Na transplanted (N=270)** | **p** |
| --- | --- | --- | --- | --- |
| Age | 56·88 ± 8·63 | 57·10 ± 8·44 | 60·44 ± 6·93 | <0·001 |
| Sex (women) | 429 (27·4%) | 90 (33·3%) | 69 (25·6%) | 0·047 |
| Height (cm) | 168·00 ± 8·97 | 167·82 ± 8·78 | 166·80 ± 8·30 | 0·173 |
| Weight (kg) | 76·97 ± 15·83 | 76·42 ± 16·72 | 74·52 ± 14·92 | 0·164 |
| Body mass index (kg/m2) | 27·24 ± 4·87 | 27·15 ± 5·33 | 26·77 ± 4·95 | 0·402 |
| Ascites (moderate-severe) | 1,012 (64·5%) | 80 (29·6%) | 214 (79·3%) | <0·001 |
| Urea (mg/dL) | 55·37 ± 36·82 | 25·07 ± 8·60 | 58·96 ± 26·82 | <0·001 |
| Creatinine (mg/dL) | 1·18 ± 0·78 | 0·64 ± 0·16 | 1·14 ± 0·31 | <0·001 |
| RFH-GFR (ml/min) | 52·25 ± 22·30 | 92·38 ± 18·65 | 45·93 ± 12·76 | <0·001 |
| International Normalized Ratio | 1·82 ± 0·64 | 1·62 ± 0·31 | 1·39 ± 0·29 | <0·001 |
| Bilirubin (mg/dL) | 5·10 (IQR 2·90-8·77) | 4·45 (IQR 3·30-6·10) | 1·70 (IQR 1·10-2·52) | <0·001 |
| Sodium (mmol/L) | 133·58 ± 5·49 | 138·47 ± 3·38 | 134·63 ± 3·89 | <0·001 |
| Albumin (g/dL) | 3·11 ± 0·65 | 2·90 ± 0·53 | 3·44 ± 0·53 | <0·001 |
| Primary outcome | 139 (8·9%) | 6 (2·2%) | 17 (6·3%) | 0·019 |

RFH-GFR: Royal Free Glomerular Filtration Rate

**Supplementary figure 1.** Bar-calibration plots of the Gender-Equity Model for liver Allocation corrected by serum sodium (GEMA-Na), the Model for End-stage Liver Disease (MELD), its sodium-corrected variant (MELD-Na) and MELD 3·0 in the overall study population of 6,071 patients. The predicted and observed probabilities for the primary outcome are presented after stratification of the study population in deciles of risk. P values correspond to the Greenwood-Nam-D’Agostino goodness-of-fit test.


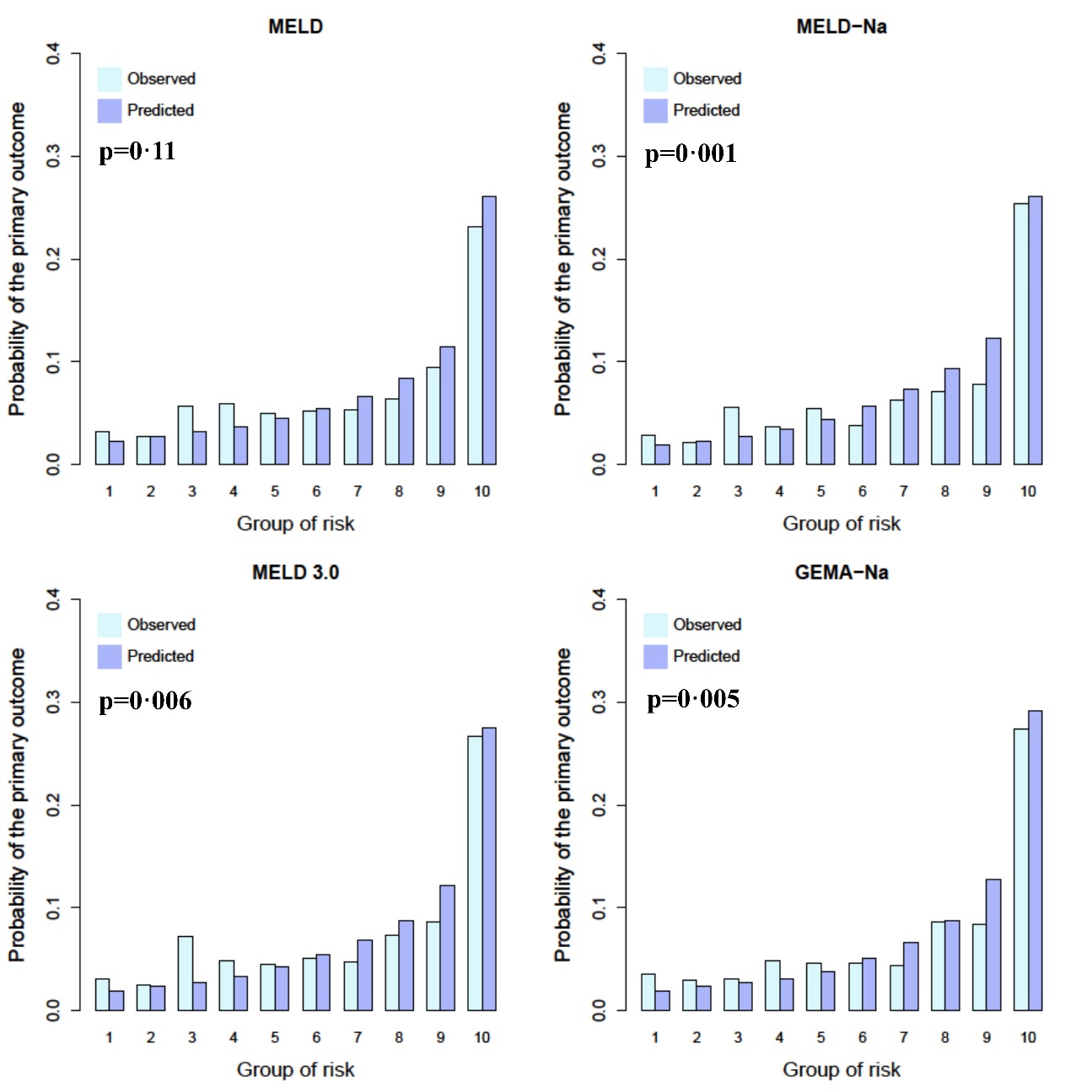


**Supplementary figure 2.** Bar-calibration plots of the Gender-Equity Model for liver Allocation corrected by serum sodium (GEMA-Na), the Model for End-stage Liver Disease (MELD), its sodium-corrected variant (MELD-Na) and MELD 3·0 in patients with indications other than tumours (n=3,606). The predicted and observed probabilities for the primary outcome are presented after stratification of the study population in deciles of risk. P values correspond to the Greenwood-Nam-D’Agostino goodness-of-fit test.


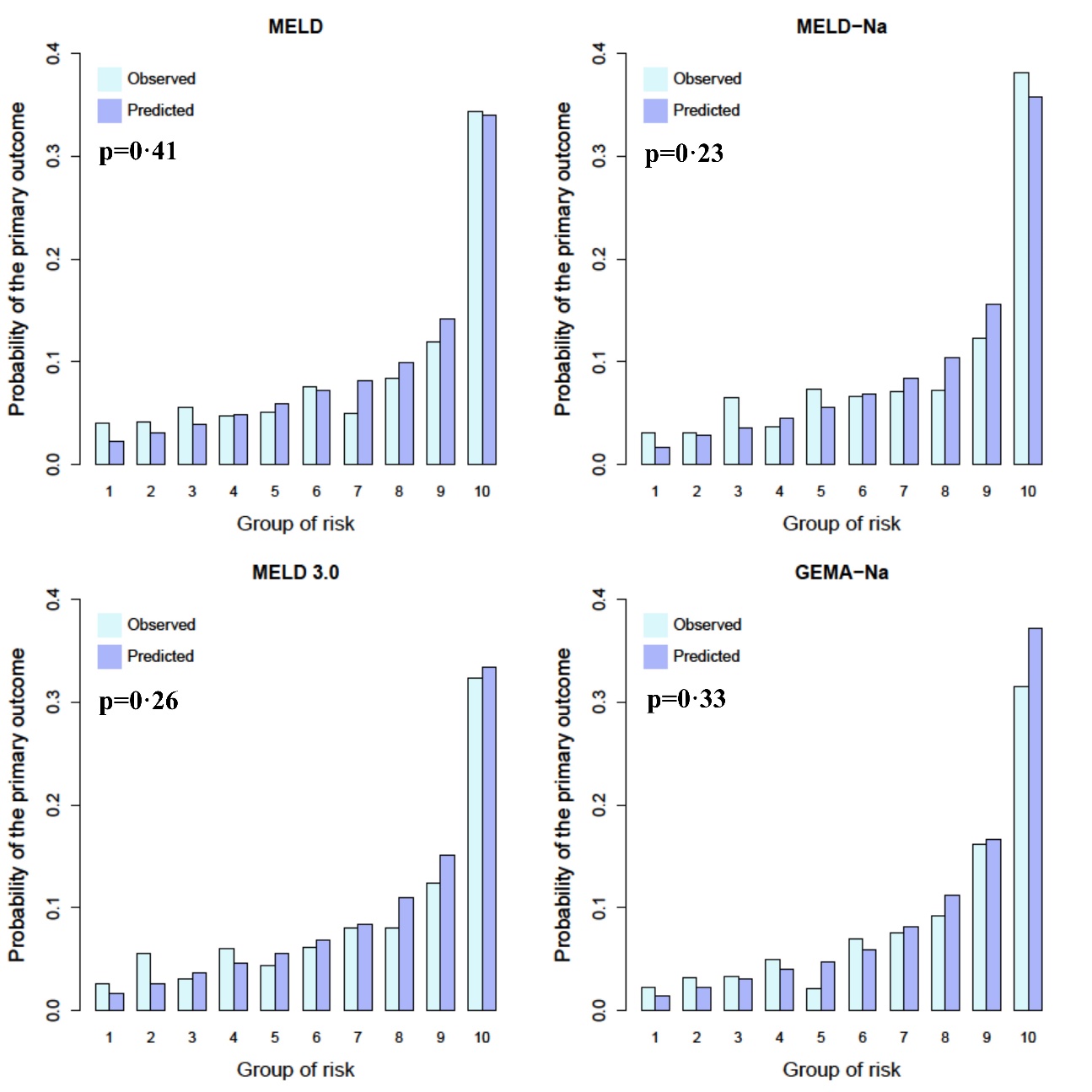


**REFERENCES**

1. Kim WR, Biggins SW, Kremers WK, et al. Hyponatremia and mortality among patients on the liver-transplant waiting list. *N Engl J Med* 2008; **359**(10): 1018-26.

2. Kim WR, Mannalithara A, Heimbach JK, et al. MELD 3.0: The Model for End-Stage Liver Disease Updated for the Modern Era. *Gastroenterology* 2021; **161**(6): 1887-95 e4.

3. Rodriguez-Peralvarez ML, Gomez-Orellana AM, Majumdar A, et al. Development and validation of the Gender-Equity Model for Liver Allocation (GEMA) to prioritise candidates for liver transplantation: a cohort study. *Lancet Gastroenterol Hepatol* 2023; **8**(3): 242-52.

4. Kalafateli M, Wickham F, Burniston M, et al. Development and validation of a mathematical equation to estimate glomerular filtration rate in cirrhosis: The royal free hospital cirrhosis glomerular filtration rate. *Hepatology* 2017; **65**(2): 582-91.

5. Jinks RC, Royston P, Parmar MK. Discrimination-based sample size calculations for multivariable prognostic models for time-to-event data. *BMC Med Res Methodol* 2015; **15**: 82.

6. Royston P, Sauerbrei W. A new measure of prognostic separation in survival data. *Stat Med* 2004; **23**(5): 723-48.
